# 5-Aza-2′-deoxycytidine reactivates the *CDH1* gene without influencing methylation of the entire CpG island or histone modification in a human cancer cell line

**Published:** 2004-07-01

**Authors:** Ken Tachibana, Ken Takeda, Masahiko Shiraishi

**Affiliations:** *) DNA Methylation and Genome Function Project, National Cancer Center Research Institute, 1-1 Tsukiji 5-chome, Chuo-ku, Tokyo 104-0045, Japan; **) Department of Hygiene-Chemistry, Faculty of Pharmaceutical Sciences, Tokyo University of Science, 2641 Yamazaki, Noda, Chiba 278-8510, Japan

**Keywords:** 5-aza-2′-deoxycytidine, *CDH1*, DNA methylation, gene silencing, histone acetylation, histone methylation

## Abstract

It is well-recognized that DNA methylation and histone modifications play critical roles in epigenetic regulation of gene activity through the alteration of chromatin structure. Recent studies have shown that in a subset of cancer cells, the silencing of the human E-cadherin (*CDH1*) gene is associated with hypermethylation of the CpG island. However, the associated molecular mechanism remains unclear. To understand the mechanism, we have investigated the alteration of CpG island methylation and histone modifications during the reactivation of the *CDH1* gene by treatment with 5-aza-2′-deoxycytidine (5-aza-dC). Although the *CDH1* gene expression was recovered by treatment with 5-aza-dC in a liver cancer cell line Li21, the methylation status of the entire CpG island and acetylation and methylation status of associated histones were not significantly altered. These results demonstrate that the silenced *CDH1* gene can be reactivated without apparent alteration of histone modification or CpG island methylation.

## Introduction

Aberrant CpG island methylation plays an important role in regulation of gene activity through alteration of chromatin structure.1) A number of studies have shown that CpG island methylation is associated with silencing of cancer-related genes in human cancer cells such as *CDKN2A*, *RB1*, *MLH1*, *MGMT* and *APC* genes.2)–6) Accumulating evidence suggests that post-translational histone modifications, such as acetylation, methylation, phosphorylation, and ubiquitination, are alternative epigenetic mechanisms in transcriptional regulation.7)–9) In particular, histone acetylation and methylation are attracting research interest. Acetylated histones H3 and H4 and methylated histone H3 at the lysine residue at position 4 (H3K4) are enriched in the euchromatic region and correlate with transcriptionally active states, whereas deacetylated histones H3 and H4 and methylated histone H3 at the lysine residue at position 9 (H3K9) are enriched in heterochromatic regions and related to transcriptionally inactive states.7)–11)

Recently, it has been shown that the processes of DNA methylation and histone deacetylation are linked by methyl-CpG binding proteins (MBPs). MeCP2 and MBD2, the members of MBPs, interact with Sin3 complex and Mi-2/NuRD complex, respectively.12),13) These complexes contain histone deacetylases (HDACs) and repress transcription of methylated genes.12),13) Moreover, it has been shown that methylation of histone H3K9 is crucial for DNA methylation in *Neurospora crassa* and *Arabidopsis thaliana*.14)–16) These data suggest functional relationships between DNA methylation and histone modification in regulation of gene activities through establishment of euchromatic or heterochromatic states.

The E-cadherin protein, a calcium-dependent homophilic cell adhesion molecule, plays a critical role in maintaining cell polarity and cell association.17) Down-regulation of the E-cadherin (*CDH1*) gene has been reported in various cancers and correlates to invasion and metastasis.18) We and others have reported that the *CDH1* gene expression is lost in some human cancer cell lines, and hypermethylation of the CpG island was observed in these cells.19)–21) However, the epigenetic mechanism of *CDH1* gene silencing is not fully understood. To investigate how DNA methylation and histone modifications regulate *CDH1* gene activity, we treated human liver cancer cell line Li21 with 5-aza-2′-deoxycytidine (5-aza-dC), a DNA methyltransferase inhibitor, and analyzed DNA methylation status and histone modifications during the reactivation of the *CDH1* gene.

## Materials and methods

### Cell culture and drug treatment

Human liver cancer cell line Li21 was maintained in RPMI 1640 medium (Invitrogen, Carlsbad, CA) supplemented with 10% fetal bovine serum (Sigma, St. Louis, MO) and 0.1 mg/ml kanamycin (Sigma) at 37 °C in a humidified 5% CO_2_ incubator. 5-Aza-dC (Sigma) was dissolved in phosphate-buffered saline, pH 6.5 [PBS (−)] at a concentration of 1 mM and stored at −80 °C in aliquots. Cells were treated with 3 μM 5-aza-dC, and fresh 5-aza-dC was replaced every 24 h.

### RT-PCR

Total cellular RNA was isolated using Tripure Isolation Reagent (Roche, Indianapolis, IN). RNA was treated with RNase-free DNase (Promega, Madison, WI) to degrade contaminated genomic DNA. Three μg of total cellular RNA was reverse transcribed with a random hexamer and M-MLV reverse transcriptase (Invitrogen) in a 40 μl of total reaction volume following the manufacturer’s recommendation. The reaction product was diluted 10-fold. PCR reactions were performed using 2 μl of diluted cDNA in a 50 μl of total reaction volume. Twenty-eight cycles of amplification (30 s at 95 °C; 30 s at 60 °C; 1 min at 72 °C) were applied for the *CDH1* gene, 35 cycles for the *Snail* (*SNAI1*) gene, and 25 cycles for the glyceraldehyde-3-phosphate dehydrogenase (*GAPDH*) gene. PCR primers used in these experiments are summarized in Table I.

### Western blot analysis

Cells were washed with PBS (−) twice, and lysed with radioimmunoprecipitation (RIPA) buffer (10 mM Tris-HCl, pH 7.5/150 mM NaCl/2 mM EDTA/1% NP-40/0.1% SDS), and lysates were stored at −80 °C until use. Proteins (10 μg per lane for *α*-tubulin and 20 μg per lane for DNMT1) were separated by SDS-polyacrylamide gel electrophoresis (PAGE), and transferred to nitrocellulose membrane. The membrane was blocked for 1 h with 5% nonfat dry milk in TBS-T (Tris-buffered saline, pH 7.6/0.1% tween-20) at ambient temperature, and probed with the following primary antibodies: anti-DNMT1 (New England Biolabs, Beverly, MA), and anti-*α*-tubulin (Oncogene research products, San Diego, CA). The membrane was then washed three times and incubated for 1 h with horseradish peroxidase-conjugated secondary antibodies. The signal was visualized with the ECL Plus detection system (Amersham Bioscience, Buckinghamshire, UK).

### Bisulfite genomic sequencing

Genomic DNA was isolated using Wizard^®^ Genomic DNA Purification System (Promega). Bisulfite modification of *Xba* I digested genomic DNA was performed following a published procedure.22) Bisulfite-treated genomic DNA was subjected to PCR experiments. Reactions were hot-started with TaqStart^TM^ antibody (BD Biosciences Clontech, Palo Alto, CA). Thirty-five cycles of amplification (30 s at 95 °C; 30 s at 53 °C; 3 min at 70 °C) were performed with AmpliTaq^®^ DNA polymerase, Stoffel Fragment (Applied Biosystems, Foster City, CA). Primers used for the PCR experiments are summarized in Table I.

### Chromatin immunoprecipitation (ChIP)

ChIP analysis of histones was performed as previously described19) with slight modification. Briefly, cells treated with 1% formaldehyde were scraped off culture dishes in PBS (−) containing 1 mM phenylmethylsulfonyl fluoride, 1 μg/ml aprotinin, 1 μg/ml pepstatin A, 1 μg/ml leupeptin, 1 μg/ml antipain, and 1 μg/ml chymostatin. Cells collected by centrifugation were lysed in SDS buffer (1% SDS/10 mM EDTA/50 mM Tris-HCl, pH 8.1) and placed on ice for 10 min. Crosslinked chromatin was sonicated to reduce the size of DNA fragments to less than 1 kb. After centrifugation, supernatant was diluted 1:10 in dilution buffer (0.01% SDS/1.1% Triton X-100/1.2 mM EDTA/16.7 mM Tris-HCl, pH 8.1/167 mM NaCl). Each antibody was added, and the mixture was incubated overnight at 4 °C with rotation. Antibodies that were used are; anti-acetyl histone H3 (06-599; Upstate Biotechnology, Lake Placid, NY), anti-acetyl histone H4 (06-866; Upstate Biotechnology), anti-monomethyl histone H3K4 (ab8895; Abcam, Cambridge, UK), anti-dimethyl histone H3K4 (ab7766; Abcam), anti-trimethyl histone H3K4 (ab8580; Abcam), anti-histone H3 C-terminus (ab1791; Abcam), anti-dimethyl histone H3K9,5),23) and rabbit IgG (F403-1; Inter-cell Technologies, Hopewell, NJ) for negative control experiments. Immune complexes were eluted (1% SDS/0.1 M NaHCO_3_, ambient temperature, 15 min twice) and crosslinks were reversed by heating (65 °C, overnight). DNA fragments were recovered from eluate by ethanol precipitation after proteinase K treatment and phenol extraction. Immunoprecipitated DNA associated with the *CDH1* gene was measured by quantitative real-time PCR using the ABI Prism^®^ 7900HT sequence detection system (Applied Biosystems). Primers and the probe used for the PCR experiments are summarized in Table I. The relative amount of histone modifications was calculated by dividing the values for modified histones by the values for histone H3 C-terminus as previously described.24)

## Results and discussion

**Fig. 1:**
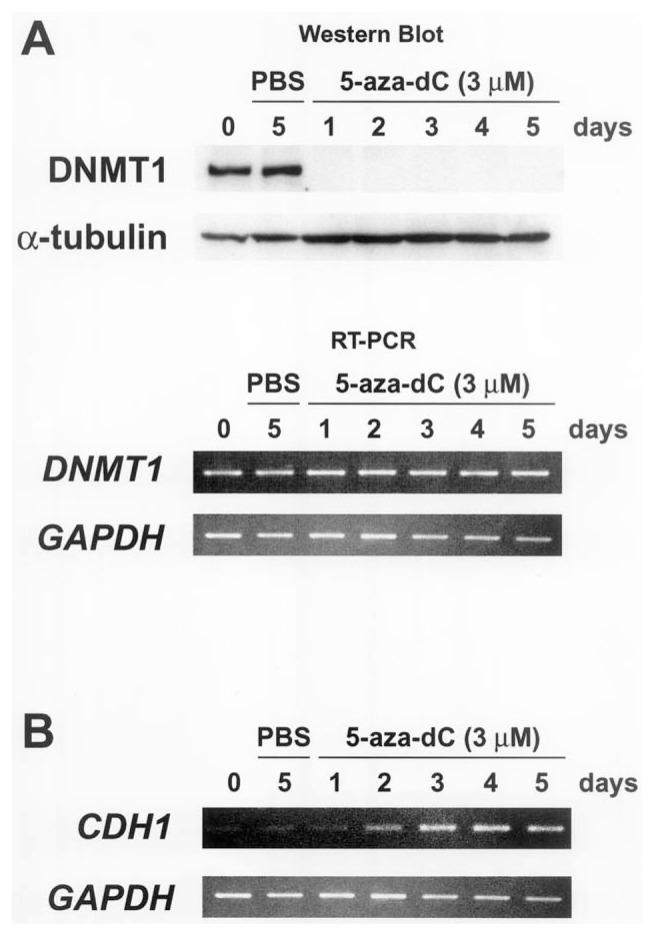
5-Aza-dC depletes DNMT1 protein and reactivates *CDH1* gene expression. Li21 cells were treated with PBS(−) or 3 μM 5-aza-dC for up to 5 days. After 5-aza-dC treatment, (A) the presence of DNMT1 protein was assessed in whole cell extracts by western blot analysis, and the expression levels of *DNMT1* mRNA were assessed by RT-PCR. (B) The expression levels of the *CDH1* mRNA after 5-aza-dC treatment were measured by RT-PCR.

The human *CDH1* gene has a CpG island containing a well-characterized promoter region.25) Previous studies have revealed that the *CDH1* gene is silenced and the CpG island is methylated in several cancer cell lines.19)–21) It is therefore predicted that CpG island methylation is associated with inactivation of the *CDH1* gene. In order to investigate how DNA methylation regulates *CDH1* gene activities, we treated the *CDH1*-silenced cancer cells with 5-aza-dC. Since the magnitude of the reactivation of the *CDH1* gene in Li21 cells was the greatest of all the investigated cell types (data not shown), we focused on this cell line. Previous studies have shown that the DNA methyltransferase covalently binds to 5-aza-dC that is incorporated into DNA and loses its enzymatic activity.26) This binding consequently results in depletion of the extractable enzyme in the cell lysate.27) Western blot analysis showed a drastic decrease in the levels of extractable DNMT1 protein at day 1 of treatment of Li21 cells, and this depletion persisted until day 5 (Fig. 1A). In contrast, the levels of DNMT1 mRNA were unaffected by drug treatment (Fig. 1A). As these results corroborate the proposed mechanism,26),27) we can speculate that 5-azadC is fully effective under these conditions. The *CDH1* gene is slightly expressed in Li21 cells without drug treatment (Fig. 1B), transcriptional reactivation became significantly apparent at day 3, and the gene expression persisted throughout the time course (Fig. 1B). From these results, we predict that the reactivation of the *CDH1* gene caused by treatment with 5-aza-dC is related to inhibition of the maintenance methylation activity of DNMT1, possibly resulting in demethylation of the CpG island.

**Fig. 2:**
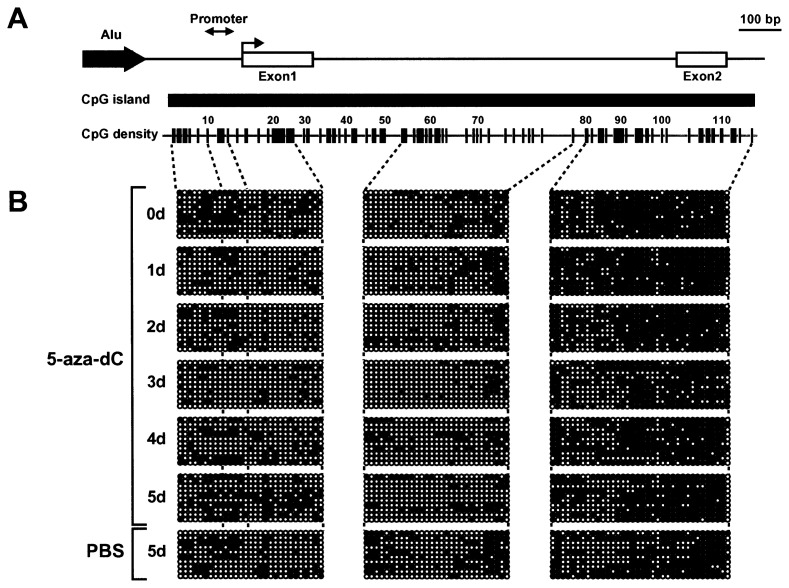
Alteration of DNA methylation status of the *CDH1* gene during treatment with 5-aza-dC. (A) Structure of the CpG island of the *CDH1* gene. The vertical lines represent the location of CpG sites. Three regions were analyzed by bisulfite genomic sequencing. (B) Methylation status of the CpG island during treatment of 5-aza-dC. After 5-aza-dC treatment, genomic DNA was extracted and the methylation status was analyzed by bisulfite genomic sequencing. Open and closed circles denote unmethylated, and methylated CpG sites, respectively. Each row indicates the specific plasmid clone.

Previous studies have shown that the treatment of cancer cell lines with 5-aza-dC causes demethylation of the CpG island and reactivation of gene expression, such as *CDKN2A* and *MLH1* genes.4),28) In addition, CpG island hypermethylation of the *CDH1* gene was partially reversed by treatment of 5-aza-dC in some cancer cells.29),30) We then investigated the DNA methylation status of the *CDH1* gene before and after treatment of 5-aza-dC in Li21 cells. Methylated CpGs were distributed densely in the 3′ region of the CpG island, while the promoter region was methylated to a lesser extent in Li21 cells (Fig. 2B). Unexpectedly, the DNA methylation status of the CpG island of 5-aza-dC treated Li21 cells was largely unaltered throughout the period of drug treatment (Fig. 2B). A similar phenomenon was observed at the *CDKN2A* locus in human colon cancer HCT116 cells lacking the *DNMT1* gene.31) These results show that depletion of the DNMT1 protein is not solely responsible for demethylation of the entire CpG island of the *CDH1* gene. Since the *CDH1* gene was reactivated under these conditions (Fig. 1B), it is suggested that demethylation of the entire CpG island is not required for reactivation of the *CDH1* gene in Li21 cells.

A previous study reported that 5-aza-dC induces histone hyperacetylation by a mechanism independent of DNA demethylation.32) Considering this observation, we investigated the alteration of histone modifications during the treatment with 5-aza-dC. In Li21 cells, histone H3 was hypoacetylated, histone H4 was hyperacetylated, and histone H3K4 and H3K9 were methylated.19) Since significant reactivation of the *CDH1* gene was observed at day 3, and this induction persisted throughout the time course (Fig. 1B), we focused on the alteration of the histone modifications of this period.

In order to perform chromatin immunoprecipitation experiments, we used an antibody against the C-terminus of histone H3 as a positive control for the binding of histone to DNA. If modified histones at the *CDH1* promoter lose DNA contact, they might be no longer crosslinkable to DNA. As a consequence, all attempts to immunoprecipitate histones from the *CDH1* promoter would fail independently of their modification status. Since no modification of the C-terminal part of histone H3 was found, it could provide a good internal control of histone binding independent of histone modifications.

**Fig. 3:**
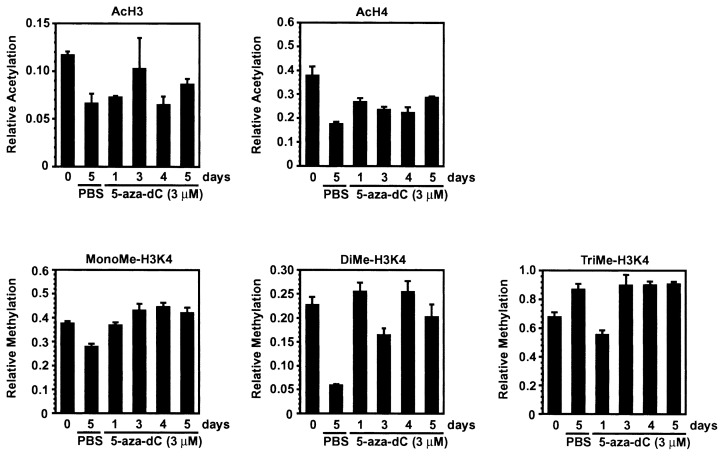
Effects of 5-aza-dC on modified histones associated with the CpG island. Li21 cells were treated with 5-aza-dC for time periods indicated, and subjected to ChIP analysis. Immunoprecipitated DNA associated with the *CDH1* gene was measured by quantitative real-time PCR. The PCR reaction was performed using specific primers and a probe for the promoter region of the *CDH1* gene. The relative amount of histone modifications was calculated as described in materials and methods. Values shown are the mean±SD of triplicate PCR reactions. Ac, acetyl; MonoMe, monomethyl; DiMe, dimethyl; TriMe, trimethyl.

Methylation of histone H3K9 was undetectable throughout the experiment (data not shown). This apparent discrepancy between the previous19) and the current study could be attributed to the use of different antibodies. It has been shown that the antibody used in this study is highly specific for methylated histone H3K9.5),23) We confirmed, by dot blot analysis using synthetic peptides, that this antibody binds to methylated histone H3K9 but not to unmethylated histone H3K9 (data not shown). These results suggest that histone H3K9 associated with the promoter region of the *CDH1* gene is not methylated in Li21 cells. Although previous studies reported that methylation of histone H3K9 at the *CDKN2A* and *MLH1* locus is reversed by treatment with 5-aza-dC,33),34) the reversal of H3K9 methylation was not observed in this study. Acetylation levels of histone H3 and H4, and methylation levels of histone H3K4 were not significantly affected by 5-aza-dC treatment (Fig. 3). These results suggest that acetylation and methylation of histones investigated in this study do not have a direct role for the reactivation of the *CDH1* gene caused by 5-aza-dC treatment.

It is reported that expression of the *SNAI1* gene, encoding a transcription factor, is inversely correlated with the expression of the *CDH1* gene, and a potential role for *CDH1* gene silencing by Snail protein has been proposed.35),36) Considering this phenomenon, we investigated the expression of the *SNAI1* gene in various human cancer cell lines. The expression of the *SNAI1* gene was detected by RT-PCR experiments regardless of the expression of the *CDH1* gene (data not shown). These results suggest that the expression of the *SNAI1* gene is not directly associated with the silencing of the *CDH1* gene.

There are a number of possible explanations for the mechanism of the reactivation of the *CDH1* gene in Li21 cells induced by 5-aza-dC. (1) There could be other CpG sites that are critical for transcriptional activity of the *CDH1* gene and demethylation of these sites may be required to reactivate transcription. (2) A very limited number of the CpG sites in the promoter region could be essential for gene reactivation, but they were not elucidated from this study. (3) 5-Aza-dC may influence histone modification, mechanisms that we have not investigated. (4) 5-Aza-dC influences the pathway involving ATP-dependent chromatin remodeling factors and alters the chromatin structure without altering epigenetic modifications.37),38) (5) 5-Aza-dC induces the expression of genes for transcription factors that are able to bind to methylated binding sites. Studies along these lines are now in progress.

## Figures and Tables

**Table I:** PCR primers and the probe used for analysis

Methods	Gene (accession no.)	Primer (positions)	Sequence (5′ to 3′)
RT-PCR	*CDH1* (NM_004360)	Forward (1216–1240)	AGTCACTGACACCAACGATAATCCT
		Reverse (1291–1315)	TTTCAGTGTGGTGATTACGACGTTA
	*DNMT1* (NM_001379)	Forward (5068–5091)	GAGGAAGCTGCTAAGGACTAGTTC
		Reverse (5250–5273)	ACTGCACAATTTGATCACTAAATC
	*GAPDH* (NM_002046)	Forward (511–530)	ATCATCAGCAATGCCTCCTG
		Reverse (848–867)	CTGCTTCACCACCTTCTTGA
	*SNAI1* (NM_005985)	Forward (522–541)	GAAAGGCCTTCAACTGCAAA
		Reverse (751–770)	TGACATCTGAGTGGGTCTGG
Bisulfite genomic sequencing	*CDH1* (AC099314)	Region 1 Forward (80624–80643)	TAGATTTTAGTAATTTTAGG
		Region 1 Reverse (80935–80954)	ACTCCAAAAACCCATAACTA
		Region 2 Forward (81147–81166)	TTTTTAGTGATGGGAGTGGG
		Region 2 Reverse (81585–81604)	ATCACCCCCTCAAAACCTAA
		Region 3 Forward (81585–81604)	TTAGGTTTTGAGGGGGTGAT
		Region 3 Reverse (82014–82033)	ACTTACCCATTACAACCCAA
ChIP analysis	*CDH1* (AC099314)	Forward (80747–80765)	TCAGCCAATCAGCGGTACG
		Reverse (80845–80866)	TCTGAACTGACTTCCGCAAGCT
		Probe (80819–80843)	ACAGGTGCTTTGCAGTTCCGACGCC
